# Leukemia inhibitory factor and its receptor: expression and regulation in the porcine endometrium throughout the estrous cycle and pregnancy

**DOI:** 10.5713/ajas.18.0429

**Published:** 2018-07-26

**Authors:** Inkyu Yoo, Soogil Chae, Jisoo Han, Soohyung Lee, Hyun Jong Kim, Hakhyun Ka

**Affiliations:** 1 Department of Biological Science and Technology, Yonsei University, Wonju 26493, Korea; 2 Dairy Science Division, National Institute of Animal Science, Rural Development Administration, Cheonan 31000, Korea

**Keywords:** Pig, Endometrium, Estrous Cycle, Pregnancy, Leukemia Inhibitory Factor, Leukemia Inhibitory Factor Receptor

## Abstract

**Objective:**

Leukemia inhibitory factor (LIF) binds to a heterodimeric receptor composed of LIF receptor (LIFR) and glycoprotein 130 (GP130) to transmit signals into the cell. LIF plays an important role in reproduction by regulating immune response, decidualization, and implantation in several species. However, the expression of *LIF* and *LIFR* in the endometrium throughout the estrous cycle and pregnancy in pigs is not fully understood.

**Methods:**

We analyzed the expression of *LIF* and *LIFR* in the endometrium on days 0 (estrus), 3, 6, 9, 12, 15, and 18 of the estrous cycle, and days 12, 15, 30, 60, 90, and 114 of pregnancy, in conceptuses on days 12 and 15, and in chorioallantoic tissues on days 30, 60, 90, and 114 of pregnancy in pigs. We also determined the effects of estrogen and progesterone on the expression of *LIF* and *LIFR* in endometrial tissues.

**Results:**

The expression of *LIF* increased in the endometrium during the late diestrus phase of the estrous cycle and during mid- to late- pregnancy, while the expression of *LIFR* increased during early pregnancy. The expression of *LIF* was induced by increasing doses of estrogen, whereas the expression of *LIFR* was induced by increasing doses of progesterone.

**Conclusion:**

These results indicate that the expression of *LIF* and its receptor *LIFR* in the endometrium is regulated in a stage-specific manner during the estrous cycle and pregnancy, suggesting that LIF and its receptor signaling system may play critical roles in regulating endometrial function in pigs.

## INTRODUCTION

Leukemia inhibitory factor (LIF) is a pleiotropic member of the interleukin-6 (IL-6) family of cytokines [1,2]. LIF regulates cellular functions by binding to a membrane-bound heterodimeric receptor, LIF receptor (LIFR) and glycoprotein 130 (GP130) [3]. Binding of LIF to LIFR and GP130 forms a high affinity functional receptor complex and activates the Janus kinase-signal transducer and activator of the transcription signaling pathway [3,4]. In addition to the well-known function of LIF in inhibiting differentiation of mouse embryonic stem cells to maintain pluripotency and self-renewal [5], LIF is involved in a variety of biological processes such as cell differentiation, bone metabolism, inflammation, vascularization, and embryogenesis [3].

In the female reproductive tract, LIF plays important roles in regulation of immune response, decidualization, and implantation in several animal species [1,3,6]. In humans, *LIF* is expressed in the endometrial glands during the luteal phase of the menstrual cycle, and *LIFR* and *GP130* are expressed in endometrial luminal epithelial cells during the cycle and in decidual stroma during early pregnancy [7,8]. LIF affects endometrial receptivity by inducing decidualization of stromal cells and increasing IL-6 and IL-15 production in decidual cells during the implantation period [9]. In mice, *Lif* is expressed in endometrial glands at the highest levels on day 4 of pregnancy [10], while *Lifr* and *Gp130* are expressed in luminal epithelial cells on days 3 to 5 of pregnancy [11]. *Lif*-null mice are infertile due to defects in implantation and decidualization [2,12], and LIF induces recruitment of various leukocytes into the site of implantation in mice [1,6]. In pigs, the expression of *LIF* and its receptor has been reported in the endometrium. *LIF* is expressed in the endometrium between days 10 and 18 of the estrous cycle and early pregnancy [13,14], and *LIFR* and *GP130* are expressed in the endometrium during the estrous cycle and pregnancy and in the placenta [15–18]. However, the expression of *LIF* and *LIFR* in the endometrium throughout all stages of the estrous cycle and pregnancy has not been determined.

The expression of *LIF* is increased by leptin, IL-1β, tumor necrosis factor-α, and transforming growth factor-β in endometrial tissues, cultured endometrial cells, and decidua in humans [1]. In mice, endometrial expression of *Lif* is induced by a nidatory surge of estrogen at implantation [10,19]. In golden hamsters, *Lif* expression is induced by estrogen while the expression of *Lifr* and *Gp130* is induced by progesterone [20]. In pigs, the expression of *GP130* is not affected by estrogen and progesterone [18]. However, the regulatory mechanism of *LIF* and *LIFR* expression in the endometrium is not fully understood in pigs. Therefore, to clarify the role of LIF in the endometrium during the estrous cycle and pregnancy in pigs, we examined the temporal and cell type-specific expression of *LIF* and *LIFR* in the endometrium throughout the estrous cycle and pregnancy and the regulation of *LIF* and *LIFR* by steroid hormones, estrogen and progesterone, in endometrial tissues.

## MATERIALS AND METHODS

### Animals and tissue preparation

All experimental procedures involving animals were conducted in accordance with the Guide for Care and Use of Research Animals in Teaching and Research and approved by the Institutional Animal Care and Use Committee of Yonsei University and the National Institute of Animal Science. Sexually mature crossbred female gilts were assigned randomly to either cyclic or pregnant status. The reproductive tracts of gilts were obtained immediately after slaughter at a local slaughterhouse on either days 0, 3, 6, 9, 12, 15, or 18 of the estrous cycle or days 12, 15, 30, 60, 90, or 114 of pregnancy (n = 3–6/d/status). Pregnancy was confirmed by the presence of apparently normal filamentous conceptuses in uterine flushings on days 12 and 15 and the presence of embryos and placenta on later days of pregnancy. Conceptus tissues were obtained from uterine flushings on days 12 and 15 of pregnancy, and chorioallantoic tissues were obtained on days 30, 60, 90, and 114 of pregnancy (n = 3–4/d).

Endometrium, dissected free of the myometrium, was collected from the middle portion of each uterine horn, snap-frozen in liquid nitrogen, and stored at −80°C prior to RNA extraction. For *in situ* hybridization, cross-sections of endometrium were fixed in 4% paraformaldehyde in phosphate-buffered saline (PBS) (pH 7.4) for 24 h and then embedded in paraffin as previously described [21]. For endometrial explant tissue cultures, endometrial tissues were obtained from sexually immature crossbred female gilts (n = 8) with no evidence of ovulation at a local slaughterhouse immediately after pigs were slaughtered.

### Total RNA extraction and reverse transcription polymerase chain reaction for *LIF* and *LIFR* cDNAs

Total RNA was extracted from endometrial, chorioallantoic, and conceptus tissues using TRIzol reagent (Invitrogen, Carlsbad, CA, USA) according to the manufacturer’s recommendations. The quantity of RNA was assessed spectrophotometrically, and the integrity of RNA was validated following electrophoresis in 1% agarose gel.

Four micrograms of total RNA from endometrial, chorioallantoic, and conceptus tissues were treated with DNase I (Promega, Madison, WI, USA) and reverse transcribed using SuperScript II Reverse Transcriptase (Invitrogen, USA) to obtain cDNAs. The cDNA templates were then diluted 1:4 with nuclease-free water and amplified by polymerase chain reaction (PCR) using Taq polymerase (Takara Bio, Shiga, Japan). The PCR conditions, sequences of primer pairs for *LIF* and *LIFR*, and expected product sizes are listed in Table 1. The PCR products were separated on 2% agarose gel and visualized by ethidium bromide staining. The identity of each amplified PCR product was verified by sequence analysis after cloning into the pCRII vector (Invitrogen, USA).

### Quantitative real-time reverse transcription polymerase chain reaction

To analyze levels of *LIF* and *LIFR* mRNAs in endometrial and chorioallantoic tissues, real-time reverse transcription (RT)-PCR was performed using the SYBR Green method with the Applied Biosystems StepOnePlus System (Applied Biosystems, Foster City, CA, USA). Complementary DNAs were synthesized from 4 μg total RNA isolated from different uterine endometrial tissues, and newly synthesized cDNAs (total volume of 21 μL) were diluted 1:4 with nuclease-free water and then used for PCR. Power SYBR Green PCR Master Mix (Applied Biosystems, USA) was used for PCR reactions. The final reaction volume of 20 μL included 2 μL of cDNA, 10 μL of 2X Master mix, 2 μL of each primer (100 nM), and 4 μL of dH_2_O. PCR conditions and sequences of primer pairs used for *LIF* and *LIFR* are listed in Table 1. The results are reported as expression relative to the level detected on day 0 of the estrous cycle after normalization of the transcript amount to the endogenous porcine ribosomal protein L 7 (*RPL7*) control by the 2^−ΔΔCT^ method [22].

### Non-radioactive *in situ* hybridization

We performed non-radioactive *in situ* hybridization as previously described [23,24] with minor modifications. Sections (5 μm thick) were rehydrated through successive baths of xylene, 100% ethanol, 95% ethanol, diethylpyrocarbonate (DEPC)-treated water, and DEPC-treated PBS. Tissue sections were boiled in citrate buffer (pH 6.0) for 10 min. After washing in DEPC-treated PBS, sections were digested using 5 μg/mL Proteinase K (Sigma, St. Louis, MO, USA) in TE (100 mM Tris-HCl, 50 mM ethylenediaminetetraacetic acid, pH 7.5) at 37°C. After post-fixation in 4% paraformaldehyde, sections were incubated twice for 15 min each in PBS containing 0.1% active DEPC and equilibrated for 15 min in 5× saline sodium citrate (SSC). The sections were prehybridized for 2 h at 68°C in hybridization mix (50% formamide, 5× SSC, 500 μg/mL herring sperm DNA, 250 μg/mL yeast tRNA; 200 μL on each section). Sense and antisense *LIF* and *LIFR* riboprobes were generated using partial cDNAs cloned into pCRII vectors by linearizing with appropriate restriction enzymes and labeling with digoxigenin (DIG)-UTP using a DIG RNA Labeling kit (Roche, Indianapolis, IN, USA). The probes were denatured for 5 min at 80°C and added to the hybridization mix. The hybridization reaction was carried out at 68°C overnight. Prehybridization and hybridization reactions were performed in a box saturated with a 5× SSC-50% formamide solution to avoid evaporation, and no coverslips were used. After hybridization, sections were washed for 30 min in 2× SSC at room temperature, 1 h in 2× SSC at 65°C, and 1 h in 0.1× SSC at 65°C. Probes bound to the section were detected immunologically using sheep anti-DIG Fab fragments covalently coupled to alkaline phosphatase and nitroblue tetrazolium chloride/5-bromo-4-chloro-3-indolyl phosphate (toluidine salt) as chromogenic substrate, according to the manufacturer’s protocol (Roche, USA).

### Explant cultures

Endometrial tissues from immature gilts were dissected from the myometrium and placed into warm phenol red-free Dulbecco’s modified Eagle’s medium/F-12 (DMEM/F-12) culture medium (Sigma, USA) containing penicillin G (100 IU/mL) and streptomycin (0.1 mg/mL) as described previously [21] with some modifications. The endometrium was minced with scalpel blades into small pieces (2 to 3 mm^3^), and aliquots of 500 mg were placed into T25 flasks with serum-free modified DMEM/F-12 containing 10 μg/mL insulin (Sigma, USA), 10 ng/mL transferrin (Sigma, USA), and 10 ng/mL hydrocortisone (Sigma, USA). Endometrial explants were cultured immediately after mincing in the presence of increasing doses of estradiol-17β (E_2_; 0, 5, 50, 500, or 5,000 pg/mL; Sigma, USA) and progesterone (P_4_; 0, 0.3, 3, 30, or 300 ng/mL; Sigma, USA) for 24 h with rocking in an atmosphere of 5% CO_2_ in air at 37°C. Explant tissues were then harvested and total RNA was extracted for real-time RT-PCR analysis to determine the effects of E_2_ and P_4_ on expression of *LIF* and *LIFR* mRNAs. These experiments were conducted using endometrial tissues from eight immature gilts.

### Statistical analysis

Data from real-time RT-PCR for *LIF* and *LIFR* expression during the estrous cycle and pregnancy were subjected to analysis of variance using the general linear models procedures of SAS (Cary, NC, USA). As sources of variation, the model included day, pregnancy status (cyclic or pregnant, days 12 and 15 post-estrus), and their interactions to evaluate steady-state levels of *LIF* and *LIFR* mRNAs, and effects of treatment and animal to evaluate effects of steroid hormones on *LIF* and *LIFR* mRNAs. Effects of day of the estrous cycle (day 0, 3, 6, 9, 12, 15, and 18) and pregnancy (day 12, 15, 30, 60, 90, and 114) in the endometrium, effects of day of pregnancy in chorioallantoic tissue (day 30, 60, 90, and 114), and effects of dose in explant culture for data from real-time RT-PCR for *LIF* and *LIFR* expression were analyzed by least squares regression analysis. Data are presented as means with standard error of the mean. A p-values less than 0.05 were considered significant.

## RESULTS

### Expression of *LIF* and *LIFR* mRNAs in the endometrium during the estrous cycle and pregnancy in pigs

**Figure 1:**
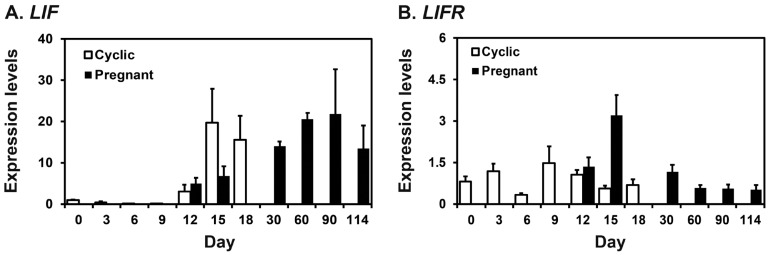
Expression of leukemia inhibitory factor (*LIF*; A) and leukemia inhibitory factor receptor (*LIFR*; B) mRNAs in the endometrium during the estrous cycle and pregnancy in pigs. Endometrial tissue samples from cyclic and pregnant gilts were analyzed by real-time reverse transcription polymerase chain reaction, and data are reported as expression relative to that detected on day 12 of the estrous cycle after normalization of the transcript amount to the endogenous ribosomal protein L7 control. Data are presented as means with standard errors.

We performed real-time RT-PCR analysis to determine whether *LIF* and *LIFR* mRNAs were expressed in the endometrium in pigs. As shown in Figure 1, real-time RT-PCR analysis showed that the expression of *LIF* in the endometrium increased at late diestrus and proestrus phases during the estrous cycle (linear effect of day, p<0.05), while the expression of *LIFR* did not change during the cycle. On days 12 and 15 post-estrus, *LIF* expression was affected by day (p<0.05), but not by pregnancy status and day×status, and *LIFR* expression was affected by pregnancy status (p<0.05) and day×status (p<0.05), but not by day. The expression of *LIFR* mRNA was greater on day 15 of pregnancy than day 15 of the estrous cycle (p<0.05). During pregnancy, the expression of *LIF* increased towards term pregnancy with the greatest expression on day 90 of pregnancy (linear effect of day, p<0.01), and whereas the expression of *LIFR* was greatest on day 15 and decreased thereafter (linear effect of day, p<0.01).

### Expression of *LIF* and *LIFR* mRNA in conceptuses during early pregnancy and chorioallantoic tissue during later stage of pregnancy

**Figure 2:**
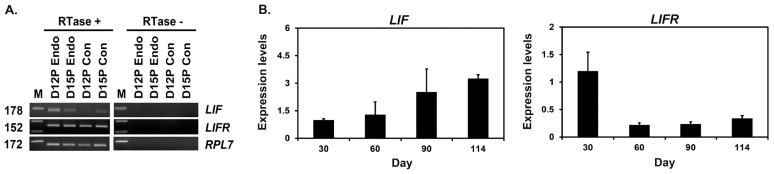
Analysis of leukemia inhibitory factor (*LIF*) and leukemia inhibitory factor receptor (*LIFR*) expression in conceptuses from day 12 and day 15 of pregnancy and chorioallantoic tissues during later stages of pregnancy. (A) Reverse transcription polymerase chain reaction (RT-PCR) analysis of *LIF* and *LIFR* mRNA in conceptuses on days 12 and 15 of pregnancy was performed using total RNA preparations. Ribosomal protein L7 (*RPL7*) was used as a positive control. RTase +/−, with (+) or without (−) reverse transcriptase; M, molecular marker; D12 Endo, endometrium on day 12 of pregnancy; D12 Con, day 12 conceptus; D15 Con, day 15 conceptus. (B) Real-time RT-PCR analysis of the expression of *LIF* and *LIFR* mRNAs in chorioallantoic tissues on days 30, 60, 90, and 114 of pregnancy. Data are reported as expression relative to that detected on day 30 of pregnancy after normalization of the transcript amount to the endogenous *RPL7* control, and data are presented as means with standard errors.

We performed RT-PCR using cDNA from conceptuses from days 12 and 15 to determine whether conceptuses express *LIF* and *LIFR* during early pregnancy. *LIF* and *LIFR* mRNAs were detected in conceptus tissues on both days of pregnancy (Figure 2A). We performed real-time RT-PCR analysis to determine if the expression of *LIF* and *LIFR* mRNAs changed in chorioallantoic tissues during mid- to late pregnancy. The expression of *LIF* mRNA increased toward term pregnancy (linear effect of day, p<0.01), whereas the expression of *LIFR* mRNA decreased after day 30 and remained low until term (linear effect of day, p<0.05) (Figure 2B).

### Localization of *LIF* and *LIFR* mRNAs in the endometrium during the estrous cycle and pregnancy in pigs

**Figure 3:**
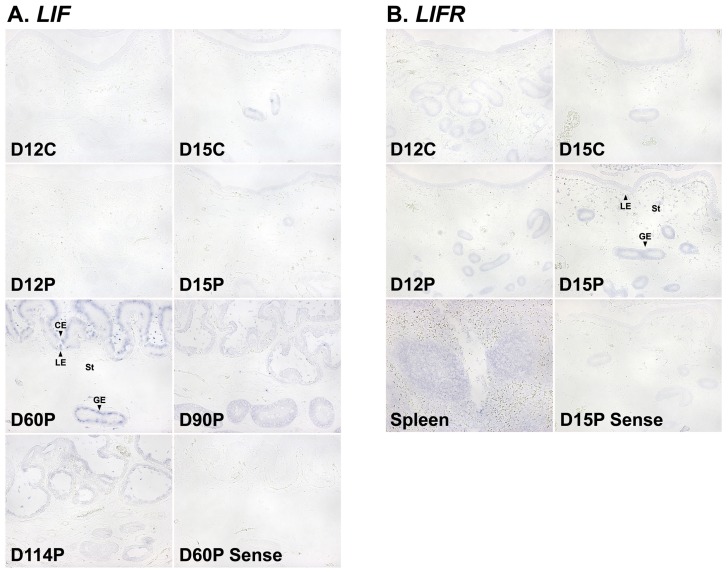
*In situ* hybridization analysis of leukemia inhibitory factor (*LIF*; A) and leukemia inhibitory factor receptor (*LIFR*; B) mRNAs in the uterine endometrium during the estrous cycle and pregnancy in pigs. Representative uterine sections from day 60 and day 15 of pregnancy, hybridized with a digoxigenin-labeled sense *LIF* and *LIFR* cRNA probe (Sense), respectively, as a negative control are shown. D, day; C, estrous cycle; P, pregnancy; LE, luminal epithelium; GE, glandular epithelium; St, stroma; CE, chorionic epithelia. Bars = 100 μm.

Next, we performed *in situ* hybridization analysis to determine which cell type(s) express *LIF* and *LIFR* mRNAs in the endometrium. The expression of *LIF* mRNA was mainly localized to endometrial luminal and glandular epithelial cells during the estrous cycle and pregnancy and to the chorioallantoic membrane during mid- to late pregnancy. *LIFR* mRNA was localized primarily to endometrial epithelial cells and stromal cells during the estrous cycle and pregnancy, with strong signal intensity in stromal cells on day 15 of pregnancy (Figure 3).

### Effects of E_2_ and P_4_ on *LIF* and *LIFR* mRNA expression in the endometrium

**Figure 4:**
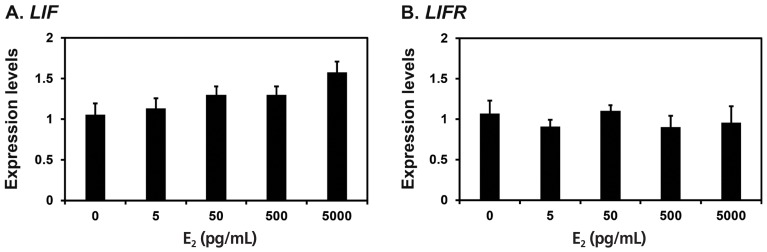
Effects of E_2_ on leukemia inhibitory factor (*LIF*) and leukemia inhibitory factor receptor (*LIFR*) mRNA levels in endometrial explant cultures. Endometrial explants from immature gilts were cultured in the presence of increasing doses of E_2_ (0, 5, 50, 500, or 5,000 pg/mL) at 37°C for 24 h. For each treatment, experiments were performed with endometria from eight gilts. Abundance of mRNA expression determined by real-time reverse transcription polymerase chain reaction analyses is relative to that for *LIF* and *LIFR* mRNAs in the control group of endometrial explants (0 pg/mL) after normalization of transcript amounts to ribosomal protein L7 mRNA. Data are presented as least squares means with standard errors.

**Figure 5:**
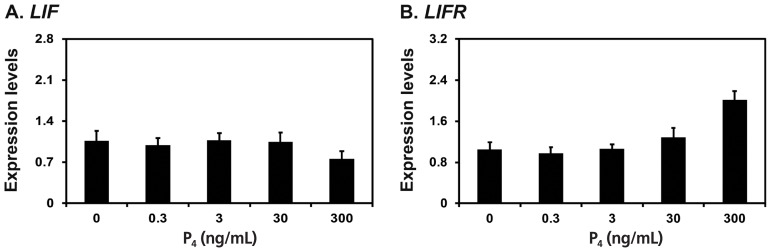
Effects of P_4_ on leukemia inhibitory factor (*LIF*) and leukemia inhibitory factor receptor (*LIFR*) mRNA levels in endometrial explant cultures. Endometrial explants from immature gilts were cultured in the presence of increasing doses of P_4_ (0, 0.3, 3, 30, or 300 ng/mL) at 37°C for 24 h. For each treatment, experiments were performed with endometria from eight gilts. Abundance of mRNA expression determined by real-time RT-PCR analyses is relative to that for *LIF* and *LIFR* mRNAs in the control group of endometrial explants (0 pg/mL) after normalization of transcript amounts to ribosomal protein L7 mRNA. Data are presented as least squares means with standard errors.

Because *LIF* mRNAs increased significantly at late diestrus and proestrus phases in this study and the expression of *Lif* and *Lifr* is induced by the steroid hormones estrogen and progesterone in rodent endometrium [19,20], we determined whether E_2_ and P_4_ affected the expression of *LIF* and *LIFR* mRNAs in endometrial explant cultures from immature gilts, that were not exposed to cyclical ovarian hormones. We treated endometrial explant tissues with 0, 5, 50, 500, or 5,000 pg/mL E_2_, and found that the expression of *LIF*, but not *LIFR*, was increased by increasing doses of E_2_ (linear effect of dose, p< 0.01) (Figure 4). Furthermore, we treated endometrial explant tissues with 0, 0.3, 3, 30, or 300 ng/mL P_4_ and found that increasing doses of P_4_ induced the expression of *LIFR* mRNA (linear effect of dose, p<0.01), but not *LIF* mRNA (Figure 5).

## DISCUSSION

The novel findings of this study in pigs are as follows: i) *LIF* and *LIFR* mRNAs are expressed in the endometrium throughout the estrous cycle and pregnancy in a stage-specific manner; ii) conceptus tissues on days 12 and 15 and chorioallantoic tissues from day 30 to term express *LIF* and *LIFR* mRNAs; iii) *LIF* and *LIFR* mRNAs are localized to epithelial and stromal cells in the endometrium; and iv) the expression of *LIF* in the endometrium is increased by E_2_, while *LIFR* is increased by P_4_. To our knowledge, this is the first report to determine the expression of *LIF* and *LIFR* throughout the estrous cycle and pregnancy in pigs.

LIF and LIFR are expressed in the endometrium, and their expression changes depending on the stage of the reproductive cycle and pregnancy, in humans and rodents. In human endometrium, *LIF* expression increases during the secretory phase of the menstrual cycle [7,8], and *LIFR* and *GP130* are expressed in epithelial cells during the cycle [8]. In mice, *Lif* expression in the endometrium is greatest on day 4 during pregnancy, which coincides with the onset of blastocyst implantation, and remains low after implantation [10]. In the present study, we found that *LIF* was expressed in the endometrium throughout the estrous cycle and pregnancy in pigs. During the estrous cycle, *LIF* expression was greatest at late diestrus and proestrus phases. These phases correspond to the period of luteolysis and follicular development, which, in turn, causes decline of progesterone and increase of estrogen. Because endometrial prostaglandin (PG) F_2α_ production is critical for the induction of luteolysis [25], we hypothesize that LIF is involved in endometrial function for the production of luteolytic PGs. In addition, because the proportion of macrophages in the endometrium significantly decreases at the time of implantation in *Lif*-null mice [26] and immune cell recruitment into the endometrium increases during the proestrus phase after luteolysis in pigs [27], LIF may be involved in regulation of immune cell recruitment into the endometrium during the estrous cycle.

LIF plays a critical role in implantation and decidualization in the uterus to increase receptivity to the developing embryo during early pregnancy in humans and rodents [1]. In pigs, the expression of *LIF* in the endometrium during pregnancy was greatest during mid- to late stages of pregnancy. Because LIF increases cell attachment and viability of porcine trophoblast cells *in vitro* [14] and was most expressed during mid- to late pregnancy, it is likely that LIF plays an important role in placental development and cell adhesion between the trophoblast and endometrial epithelial cells during mid- to late stages of pregnancy. The endometrial expression of *GP130*, which forms a heterodimeric LIF receptor with LIFR, also increases during mid-pregnancy with the greatest expression on day 60 of pregnancy in pigs [18]. Based on the expression pattern of endometrial *LIF* during pregnancy, it is likely that the function of LIF for conceptus implantation in pigs is not as critical as in humans and rodents. This may be due to differences in implantation and placentation in pigs, which forms a non-invasive true epitheliochorial type placenta and does not require a decidualization process during the implantation period, whereas in humans and rodents the endometrium is decidualized for implantation and an invasive hemochorial type placenta is formed when the embryo is implanted.

The expression of *LIFR* in the endometrium has been shown in humans [7,8], rodents [20], and pigs [16,17], but the temporal pattern of *LIFR* expression throughout the menstrual or estrous cycle and pregnancy has not been studied in any species so far. In this study, we found that the expression of *LIFR* in the endometrium was constitutive throughout the estrous cycle in pigs, but changed during pregnancy with the greatest abundance on day 15. These findings suggest that *LIFR* and *GP130* are both expressed in the endometrium, but their endometrial expression during pregnancy is differentially regulated in pigs.

Porcine conceptus tissues expressed *LIF* and *LIFR* during early pregnancy, and chorioallantoic tissues also expressed *LIF* and *LIFR* during mid- to late pregnancy. These data coincide with previous reports that *LIF* and *LIFR* are expressed in the conceptus and fetal membrane tissues [14,15]. We further determined that *LIF* expression in chorioallantoic tissues increased towards term pregnancy in pigs, while *LIFR* expression decreased, suggesting a role of LIF in placental function. Based on localization of *LIF* and *LIFR* in the endometrium and conceptus tissues, it is likely that LIF acts on endometrial epithelial cells and conceptus/chorioallantoic tissues in an autocrine and/or paracrine manner to affect endometrial and conceptus function during pregnancy in pigs.

*LIF* is expressed in endometrial epithelial cells during the menstrual cycle and endometrial epithelial cells, decidual cells, and decidual leukocytes during early pregnancy in humans [8,28]. *LIFR* is expressed in only endometrial luminal and glandular epithelial cells during the cycle, whereas it is expressed in endometrial epithelial cells, endothelial cells, and villous and extravillous trophoblasts during pregnancy in humans [8,28]. In this study, *LIF* expression was detected primarily in endometrial epithelial cells during the estrous cycle and pregnancy and in chorioallantoic membrane during mid- to late pregnancy in pigs. *LIFR* expression was also detected in endometrial epithelial cells in the endometrium, and endometrial stromal cells expressed *LIFR* at increased levels on day 15 of pregnancy, which is the time when many immune cells such as T cells and NK cells are recruited into the endometrium during pregnancy in pigs [29]. These data suggest that LIF and LIFR act on endometrial epithelial cells in an autocrine manner and on stromal cells in a paracrine manner.

The expression of *LIF* is increased by cytokines, leptin, and estrogen in endometrial cells in humans and rodents [1,10,19], and the expression of *Lifr* is induced by P_4_, but not by E_2_, in the endometrium of golden hamsters [20]. Based on the patterns of *LIF* and *LIFR* expression in the endometrium during the estrous cycle and pregnancy observed in this study and reports in other species [1,10,19,20], we hypothesized that ovarian steroid hormones affect the expression of *LIF* and *LIFR* in the porcine endometrium. Indeed, our results in this study indicate that estrogen increased the expression of *LIF* and progesterone increased the expression of *LIFR* in endometrial tissues. These data suggest that estrogen and progesterone play critical roles in regulating the expression of *LIF* and *LIFR* in the endometrium during the estrous cycle and pregnancy, and that the regulation of endometrial *LIF* and *LIFR* expression by steroid hormones is similar among species. In mice, the expression of *Lif* dramatically increases in the endometrium at the time of implantation, and estrogen is responsible for the increase of endometrial *Lif* expression [10,19]. In pigs, conceptus implantation initiates on day 12 of pregnancy and the implanting conceptus produces estrogen, which acts as a maternal pregnancy recognition signal [25]. Thus, we postulated that endometrial expression of *LIF* increased in response to conceptus-derived estrogen in pigs. However, our results showed that *LIF* expression was not different between day 12 of the estrous cycle and pregnancy. This suggests that conceptus-derived estrogen may not be responsible for endometrial expression of *LIF* in pigs, and that the regulatory mechanism underlying endometrial *LIF* expression is different between rodents and pigs because of differences in implantation and placentation.

We also found that the endometrial expression of *LIFR* was greatest on day 15 of pregnancy, when the implanting conceptus produces the large amounts of cytokines such as interferons (IFNs), IFN-γ, and IFN-δ [25], and the endometrium produces cytokines and chemokines such as cysteine-X-cysteine motif chemokine ligand (CXCL) 9, CXCL10, and CXCL11 [29]. Thus, it is possible that these cytokines derived from the conceptus and the endometrium affect the expression of endometrial *LIFR* at the time of implantation in pigs, but further study of the factors regulating endometrial *LIFR* expression on day 15 of pregnancy are necessary to elucidate this hypothesis.

In conclusion, in this study we showed that *LIF* and *LIFR* are expressed in the endometrium in a stage-specific manner during the estrous cycle and pregnancy and in conceptus and chorioallantoic tissues during pregnancy in pigs. Estrogen and progesterone regulate the expression of *LIF* and *LIFR* in the endometrium, respectively. Although the role of LIF in the endometrium during the estrous cycle and pregnancy is not fully understood, our results indicate that LIF and its receptor signaling system play important roles in regulating endometrial function during the estrous cycle and at the maternal-conceptus interface during mid- to late-pregnancy in pigs.

## Figures and Tables

**Table 1:** Summary of primer sequences for reverse transcription polymerase chain reaction (RT-PCR) and real-time RT-PCR and expected product sizes

Primer	Sequence of forward (F) and reverse (R) primers (5′→3′)	Annealing temperature (°C)	Product size (bp)	GenBank accession no.
For RT-PCR and real-time PCR				
*LIF*	F: TCA ATC CTG GTG CTG TGA AC	60	178	NM_214402.2
	R: CAG CCC AGC TTC TTC TTC TG			
*LIFR*	F: GGT CGC AAA GAG TGG AGT GA	60	163	XM_021076925.1
	R: TTC TGC CAA TCT GTG CCG AT			
*RPL7*	F: AAG CCA AGC ACT ATC ACA AGG AAT ACA	60	172	NM_001113217
	R: TGC AAC ACC TTT CTG ACC TTT GG			
For *in situ* hybridization				
*LIF*	F: GTC ACC CAT GTC ACA GCA AC	60	445	NM_214402.2
	R: CAG CCC AGC TTC TTC TTC TG			
*LIFR*	F: TCC CGA CAA TTT TTG ATT CC	60	195	XM_021076924.1
	R: TCA CAG GAT CCC TCC AAG AC			

*LIF*, leukemia inhibitory factor; *LIFR*, leukemia inhibitory factor receptor; *RPL7*, ribosomal protein L7.
